# Bayesian Conditional GAN for Unsupervised Anomaly Detection in Structural Health Monitoring Time-Series Dataset

**DOI:** 10.3390/s26134253

**Published:** 2026-07-04

**Authors:** Yohannes L. Alemu, Christian Walther, Manuel Schneider, Norbert Greifzu, Leon Quinten Thiebes, Andreas Wenzel, Uwe Plank-Wiedenbeck, Tom Lahmer

**Affiliations:** 1 Institute of Structural Mechanics, Bauhaus University, 99423 Weimar, Germany; christian.walther@uni-weimar.de (C.W.); tom.lahmer@uni-weimar.de (T.L.); 2 Faculty of Electrical Engineering, Schmalkalden University of Applied Sciences, 98574 Schmalkalden, Germany; m.schneider@hs-sm.de (M.S.); n.greifzu@hs-sm.de (N.G.); a.wenzel@hs-sm.de (A.W.); 3 Chair of Transport System Planning, Bauhaus-University Weimar, 99423 Weimar, Germany; leon.thiebes@uni-weimar.de (L.Q.T.); uwe.plank-wiedenbeck@uni-weimar.de (U.P.-W.)

**Keywords:** Bayesian inference, conditional GAN, temporal causal networks, unsupervised anomaly detection, structural health monitoring, uncertainty quantification, Bayesian conditional deep convolutional generative adversarial network

## Abstract

Detecting rare structural damage without labeled fault data remains a critical unsolved challenge in structural health monitoring (SHM). Prestressed concrete catenary poles are key elements of high-speed railway infrastructure, and undetected degradation can compromise safety and service reliability. This paper introduces BcDCGAN, a Bayesian conditional deep convolutional generative adversarial network designed for unsupervised anomaly detection in multivariate vibration time series from three in-service catenary poles. Trained exclusively on healthy acceleration signals with wind-speed conditioning, the model learns the normal structural dynamics and produces an uncertainty-based anomaly score that combines reconstruction quality, adversarial evaluation, and epistemic uncertainty into a single decision function. An adaptive, data-driven threshold estimate from healthy validation data enables practical deployment without damage labels. On a real 2017 catenary pole dataset (1606 signals, 70/10/20 split) with injected, physically motivated damage-like patterns, BcDCGAN achieves high anomaly recall with interpretable uncertainty signals and clear separation between normal and anomalous latent representations. The results suggest that Bayesian conditional GANs can support risk-aware monitoring of railway infrastructure under varying environmental and operational conditions.

## 1. Introduction

Detecting anomalies in time-series data is critical for safety-critical infrastructure, where undetected faults can lead to catastrophic failures and substantial economic losses [1,2]. The goal is to identify deviations from normal operational behavior using continuous sensor streams, ideally without prior knowledge of failure modes.

However, real-world structural health monitoring (SHM) applications face significant challenges: (i) non-stationary and noisy sensor signals influenced by varying environmental and operational conditions, (ii) complex long-range temporal dependencies that violate independent and identically distributed (i.i.d.) assumptions, (iii) extreme rarity of anomalies with fewer than 0.1% of samples, and (iv) complete absence of labeled fault examples during training [3]. These factors render traditional supervised methods impractical and cause classical unsupervised techniques such as distance-based approaches, statistical thresholding, or one-class classifiers to degrade substantially in dynamic SHM environments, as reported in recent surveys on time-series anomaly detection [4] and confirmed by the baseline results presented in Section 2.2.

In addition, purely discriminative models do not provide a measure of confidence, making them unsuitable for high-stakes decisions in structural health monitoring. Uncertainty quantification (UQ) is increasingly emphasized in regulatory discussions, such as the EU AI Act, as important for trustworthy high-risk AI systems [5].

Generative adversarial networks (GANs) have emerged as powerful tools for unsupervised anomaly detection by learning the distribution of normal data. Anomalies are flagged via high reconstruction error or low discriminator confidence [6]. However, standard GAN-based methods struggle with non-stationary multivariate time series and provide no explicit measure of epistemic uncertainty. Related readings can be found in [7,8].

Consequently, there is an urgent demand for a unified architecture that simultaneously (i) operates fully unsupervised on raw, unlabeled multivariate time series; (ii) captures complex temporal dynamics via dilated convolutions; (iii) incorporates Bayesian uncertainty modeling throughout the network; and (iv) delivers probabilistic anomaly scores and enables structural health monitoring in the complete absence of failure examples.

This paper makes the following contributions to unsupervised anomaly detection for structural health monitoring of time-series signals:

- We propose a Bayesian conditional deep convolutional GAN (BcDCGAN) architecture for multivariate vibration-based SHM, with variational weight distributions in both generator and critic to provide explicit epistemic uncertainty.
- We integrate Bayesian temporal causal networks and environmental conditioning (wind/temperature features) into the adversarial framework, specifically tailored to non-stationary SHM signals from prestressed concrete catenary poles.
- We introduce and evaluate a validation-based, multi-component Bayesian anomaly scoring and thresholding scheme on a real catenary pole dataset with injected anomalies, demonstrating deployment-ready performance with high precision and recall.

The remainder of the paper is organized as follows: Section 2 reviews related work, Section 3 motivates the approach, Section 4 describes the methodology, Section 5 presents the case study and results, and Section 6 concludes with future directions.

## 2. Literature Review

### 2.1. Time-Series Anomalies

Time-series anomalies are broadly classified into three main types: point anomalies, contextual anomalies, and collective anomalies [9,10]. Point anomalies refer to individual data points that deviate significantly from the rest of the data. Contextual anomalies are data points that are anomalous only within a specific context or time frame, such as a temperature spike that is unusual for a given season. Collective anomalies involve a sequence or group of data points that are individually unremarkable; together, however, they represent an unusual pattern, making them more complex and challenging to detect compared to point or contextual anomalies.

**Figure 1:**
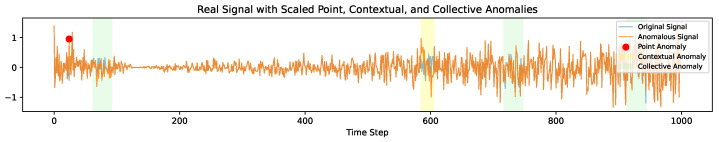
Types of time-series anomalies.

Figure 1 illustrates these types of anomalies using a representative acceleration signal segment of the catenary pole dataset used in this study [11]. Point, contextual, and collective anomalies are injected into the healthy signal segment that result in an anomalous segment. Detecting collective anomalies is often the most challenging, as individual points may not exhibit clear deviations when viewed in isolation.

### 2.2. Traditional Anomaly Detection Limitations

Traditional anomaly detection methods including distance-based approaches (Euclidean distance, KNN), statistical thresholding (moving averages, standard deviation), dimensionality reduction (PCA), and density estimation (GMM, one-class SVM) struggle with non-stationary multivariate time series common in SHM. These methods assume stationarity, linearity, or simple distributional forms that fail to capture complex temporal dynamics and environmental variations in structural vibration data.

In addition to these reported limitations, we quantitatively evaluated representative traditional and low-capacity unsupervised baselines on the real catenary pole dataset used in this study. Under a consistent validation-based thresholding scheme (mean-plus-kσ on healthy validation signals, applied unchanged to the held-out test set), a simple statistical RMS + μ+kσ rule, a nonlinear Kernel Principal Component Analysis (KPCA) reconstruction-error method, and a 1D CNN/TCN autoencoder all detect only a small fraction of injected anomalies and exhibit modest discriminative ability (Table 1). This empirical evidence further substantiates that such methods perform poorly on non-stationary SHM time series, motivating the need for more expressive generative models.

The results in Table 1 show that all three baseline methods struggle to detect the injected anomalies reliably on the catenary pole dataset used in the case study. Despite moderate precision, their low recall and F1-scores, together with ROC-AUC values around 0.70, indicate limited discriminative ability compared with the proposed BcDCGAN on the same data.

### 2.3. Generative Adversarial Network

Generative adversarial networks (GANs) Equation (1) provide a natural framework for unsupervised anomaly detection in SHM time series by learning the distribution of healthy signals and flagging deviations that are poorly reproduced by the generator or judged unrealistic by the critic. In this work, we adopt a Wasserstein GAN formulation with a conditional structure and temporal causal blocks, building on standard GAN theory rather than re-deriving it; we briefly recall the key idea of the *min max* game,

(1) $$\min_{G}\max_{D}V\left(D,G\right)=E_{x∼p_{\mathrm{data}}}\left[\log D\left(x\right)\right]+E_{z∼p_{z}}\left[\log\left(1-D\left(G\left(z\right)\right)\right)\right],$$

and refer the reader to [9,12,13] for full details.

GAN-based approaches thus address many limitations of traditional methods, particularly in capturing long-range temporal dependencies and high-dimensional distributions without requiring labeled anomalies.

### 2.4. GAN-Based Anomaly Detection Approaches

Several specialized GAN variants have been proposed for unsupervised anomaly detection (AD) in time series [14]. These approaches capitalize on the GAN’s ability to model the distribution of normal data while producing higher reconstruction errors or lower discriminator confidence for anomalous samples.

TAnoGAN introduces an LSTM-augmented GAN to learn compact latent representations of normal time series through adversarial training [15]. The anomaly scoring is based on a combination of reconstruction error and latent-space deviation. This method effectively captures sequential dependencies without requiring labeled faults.

The DCGANs + Bi-LSTM framework integrates deep convolutional GANs with bidirectional LSTM to jointly exploit spatial and long-range temporal features [16]. The convolutional generator creates realistic sequences, while the Bi-LSTM provides enhanced bidirectional context, leading to superior performance on complex multivariate signals.

BiGAN extends the conventional GAN by jointly training an encoder along with the generator and discriminator, thereby learning a bidirectional mapping between data and latent space [17]. This architecture yields more accurate reconstructions and improved anomaly separation, as outliers typically exhibit poor inverse mappings to the learned normal manifold.

These GAN-based methods Table 2, substantially advance over traditional approaches in complex time-series tasks [4]. However, they commonly lack inherent uncertainty quantification and struggle with strong non-stationarity limitations directly addressed by the Bayesian conditional formulation presented in this work.

### 2.5. Anomaly Detection Metrics

In unsupervised anomaly detection, quantitative evaluation remains challenging due to the absence of labeled anomalies in both training and real-world deployment. Various thresholding strategies are therefore applied to separate normal from anomalous samples. Common approaches include fixed thresholds, percentile-based methods (e.g., flagging the top 5% highest-scoring samples as anomalies), or statistical thresholds such as mean plus *k* standard deviations (μ+kσ, with k=2 frequently used) [2].

In adversarial models, discriminator scores near 0.5 often signal uncertainty and are interpreted as potential anomalies [4]. Reconstruction-based methods primarily use reconstruction error (e.g., mean squared error between input and reconstructed signal) as the primary anomaly indicator: a higher error suggests deviation from the learned normal distribution.

Combined scoring, which merges reconstruction error and discriminator confidence, provides a more robust signal in many GAN-based frameworks [18]. Since ground-truth labels are not available during training, recall is widely considered to be the most meaningful metric when synthetic anomalies are injected solely into the held test set [15]. Recall, given by TP/(TP+FN), measures the proportion of injected anomalies correctly identified and remains critical in safety-critical applications like structural health monitoring, where missed faults carry high risk. At the same time, precision and the associated false-alarm rate are equally important in practice because unnecessary interventions and repeated alarms are costly; therefore, both recall and precision must be considered jointly when assessing anomaly detectors for SHM.

Table 3 summarizes the evaluation strategies. When anomalies are injected exclusively into the test set, recall offers a reproducible measure of detection capability, but for SHM it must be interpreted together with precision, false-alarm rate, and threshold-dependent metrics such as ROC-AUC or PR-AUC to capture the full trade-off between safety and operational cost.

## 3. Motivation

Building on the challenges outlined in the introduction (rarity of anomalies, non-stationarity, lack of labels, and the need for uncertainty quantification), this section motivates the specific architectural choices of BcDCGAN for SHM [1,3].

Existing GAN-based anomaly detection methods (e.g., TAnoGAN, DCGAN+Bi-LSTM, BiGAN) have shown that adversarial generative models can effectively learn the distribution of normal time series and flag deviations as anomalies. However, these architectures typically operate with deterministic weights, do not natively provide explicit epistemic uncertainty, and rarely incorporate environmental conditioning or temporal causal structures explicitly optimized for non-stationary SHM signals. The proposed BcDCGAN addresses this gap by combining conditional adversarial training, Bayesian temporal causal networks, and an uncertainty-based anomaly score tailored to vibration data from prestressed concrete catenary poles [19].

The proposed Bayesian conditional deep convolutional GAN (BcDCGAN) extends this line of work by combining conditional adversarial training, temporal causal networks, and Bayesian weight distributions within a unified SHM-specific framework. In contrast to purely deterministic GANs, BcDCGAN provides explicit estimates of epistemic uncertainty through variational Bayesian inference on the generator and critic weights, and it employs an adaptive anomaly score that jointly reflects reconstruction quality, critic evaluation, and parameter uncertainty. This design is motivated by the need to improve robustness under non-stationary operating conditions and to offer more interpretable, uncertainty-based anomaly scores for vibration-based monitoring of prestressed concrete catenary poles.

## 4. Methodology

The primary objective of the proposed framework is to identify structural anomalies under varying environmental and operational conditions without prior exposure to damage-state data. To achieve this, we adopt a fully unsupervised approach centered on a Bayesian conditional deep convolutional generative adversarial network (BcDCGAN).

**Figure 2:**
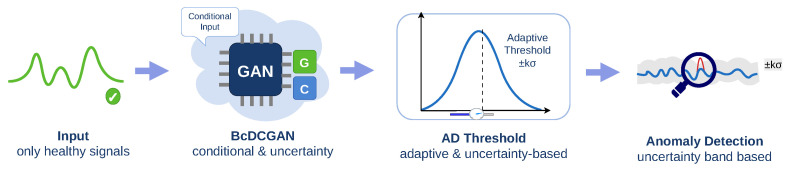
Flowchart for the proposed anomaly detection architecture.

The BcDCGAN architecture integrates variational Bayesian inference into both the generator and the critic, allowing the model to learn the underlying distribution of healthy signals while explicitly accounting for environmental and operational inputs. The general framework is illustrated in Figure 2.

After training, an adaptive threshold is estimated using a held-out validation set of healthy signals by synthesizing three distinct indicators: the reconstruction error, the critic’s evaluation score, and the epistemic uncertainty.

During deployment, incoming signals are evaluated against this adaptive threshold alongside a corresponding uncertainty band to support decision-making.

### 4.1. Bayesian Inference

Bayesian inference provides a principled framework for uncertainty quantification in deep generative models by treating network parameters as probability distributions rather than fixed values [20]. This enables the model to capture epistemic uncertainty, improving robustness in data-scarce or non-stationary SHM scenarios [21].

(2) $$P\left(H|E\right)=\frac{P(E|H)\;P(H)}{P(E)}.$$

where

P(H∣E) is the posterior probability, representing the updated belief in a hypothesis *H* after observing evidence *E*;

P(H) is the prior probability, expressing the initial belief before seeing any data;

P(E∣H) is the likelihood, indicating how likely the observed data are under hypothesis *H*;

P(E) is the evidence or marginal likelihood, serving as a normalizing constant to ensure the posterior is a valid probability distribution.

Exact posterior inference P(θ∣D) is intractable for deep networks, so variational inference (VI) approximates it with a tractable distribution q(θ) by minimizing the Kullback–Leibler (KL) divergence between the approximate and true posterior [22]. This is equivalent to maximizing the Evidence Lower Bound (ELBO), a tractable lower bound on the log-marginal likelihood that balances reconstruction accuracy and regularization:

(3) $$\mathrm{ELBO}\left(q\right)=E_{q(\theta )}\left[\log p(D|\theta )\right]-\mathrm{KL}\left(q(\theta )\|p(\theta )\right).$$

In this work, the variational posterior q(θ) is modeled as independent Gaussian distributions over the weights of the generator and critic networks. Standard Gaussian priors p(θ)=N(0,1) are placed on these weights, while the encoder remains deterministic, with no prior distribution [22]. With both the posterior and prior chosen as Gaussians, the KL divergence term in Equation (3) can be computed analytically using the closed-form expression

(4) $$\mathrm{KL}\left(N\left(\mu ,\sigma^{2}\right)\|N\left(0,1\right)\right)=\frac{1}{2}\left(\mu^{2}+\sigma^{2}-1-2\log\sigma \right).$$

where

μ is the posterior mean, representing the learned central value of the weight distribution;

σ is the posterior standard deviation, capturing the uncertainty around the mean;

logσ is the logarithmic standard deviation parameterized during training to ensure positivity.

In this work, we adopt independent Gaussian priors over the weights of the Bayesian TCN layers, following standard practice in Bayesian neural networks. Factorized priors provide a tractable baseline and avoid imposing arbitrary correlations across filters or timesteps. The variational posterior q(θ) is initialized with $\mu_{\mathrm{init}}=0.15$ and $\log\sigma_{\mathrm{init}}=1.5$ at the start of training; the KL divergence is always computed against the fixed standard normal prior p(θ)=N(0,1), which remains unchanged throughout optimization. The analytical KL term is computed for q(θ)=N(μ,exp(2logσ)) against p(θ)=N(0,1), and it is normalized by the number of parameters per layer so that per-layer KL contributions remain in a stable range. Its influence during training is further controlled via a KL warm-up schedule and the global coefficient $\beta_{\mathrm{KL}}$.

During training, the variational parameters μ and logσ are optimized jointly with the network weights using the reparameterization trick and Monte Carlo estimates of the ELBO [23]. The KL divergence term is added to the overall loss, acting as a regularizer that encourages the posterior to remain close to the prior. This optimization yields distributional weights that propagate uncertainty through forward passes and, via posterior Monte Carlo sampling during inference, provide explicit epistemic uncertainty estimates for anomaly scoring, addressing a key limitation of deterministic GANs.

This Bayesian treatment improves generalization to novel structural conditions and environmental variations, while the ELBO objective ensures stable adversarial training with meaningful latent representations [21].

### 4.2. Temporal Causal Networks

Temporal causal networks (TCNs) are convolutional architectures specifically designed for sequence modeling tasks [24]. They effectively capture long-range temporal dependencies in time-series data while maintaining computational efficiency and causal structure, making them well suited for real-time analysis of non-stationary signals such as structural vibrations.

Two key mechanisms enable this capability:

Causality: In TCNs, the output at any timestep depends only on current and past inputs, never on future values. This is achieved through causal (zero-padded) convolutions that preserve temporal order and prevent information leakage. Causality is essential for streaming applications, allowing the model to process signals sequentially as they arrive, critical for online anomaly detection in SHM.

Dilation: Dilated convolutions introduce gaps between kernel elements, exponentially expanding the receptive field with network depth without increasing parameters or losing resolution. By stacking layers with increasing dilation rates (e.g., d=1,2,4,8), TCNs efficiently aggregate information across distant timesteps, enabling the modeling of complex long-term patterns common in wind-induced or fatigue-related vibrations.

**Figure 3:**
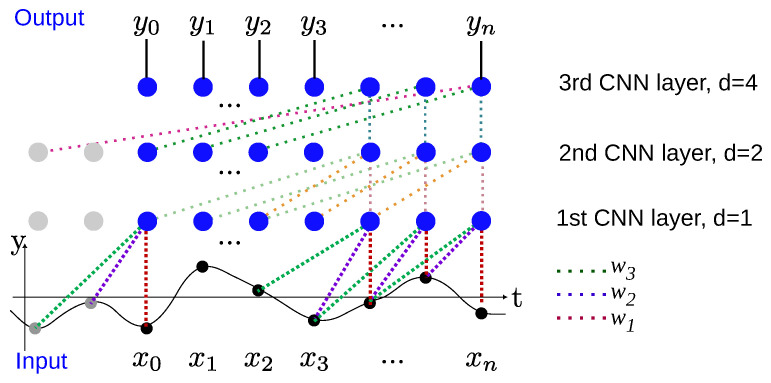
Schematic representation of a causal TCN architecture.

Figure 3 illustrates a typical causal TCN with dilation. In the proposed BcDCGAN, TCN blocks with residual connections and dilated convolutions replace standard layers in both the generator and the critic, ensuring stable training and robust temporal feature extraction in multivariate acceleration signals.

### 4.3. Proposed Bayesian Conditional Deep Convolution GAN Anomaly Detection Architecture

The proposed architecture integrates Bayesian inference into the convolutional layers of the generator and critic [22]. Weight and bias distributions are used and optimized throughout model training, enabling inclusion and quantification of uncertainty. The generator is updated from critic feedback indirectly and reconstruction error directly. An encoder extracts latent space features to help the generator produce signals with minimal error. Gradients from the generator’s loss function flow back through the generator to update the encoder, producing a latent space that is optimized to support the generator’s task. Conditional inputs such as temperature and wind speed can be fed to model components for improved context identification.

**Figure 4:**
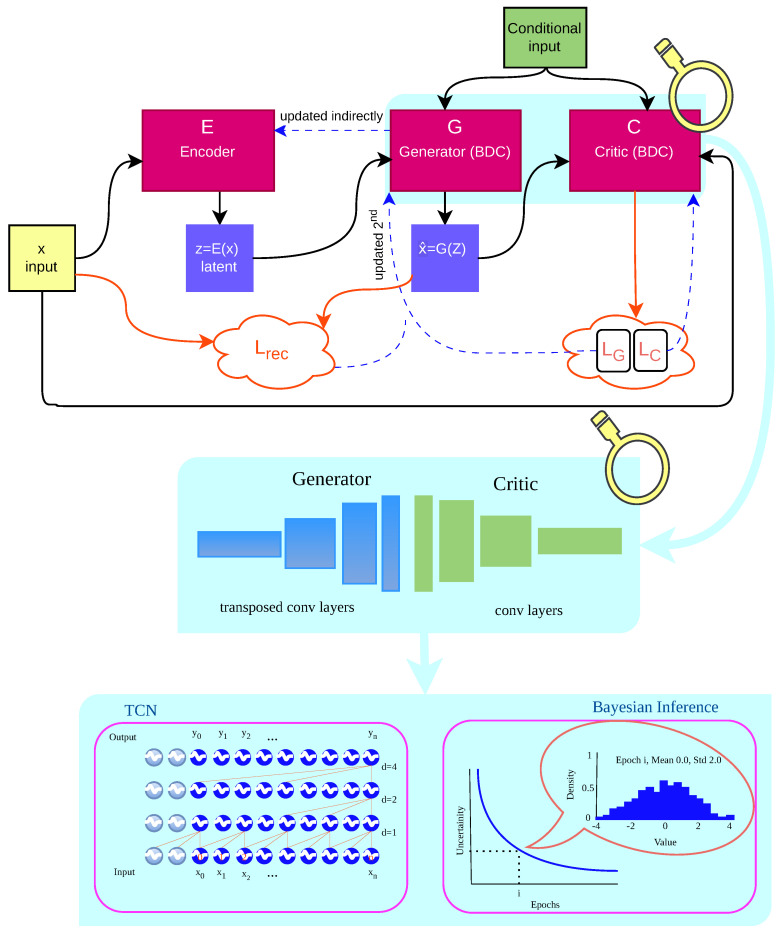
Bayesian conditional deep convolution GAN architecture.

Figure 4 shows how the generator and critic are structured. Within the generator and critic, each convolution layer operates based on the TCN, considering time-series signals up to the current sequence with selected dilation rates. This is applied to each weight and bias distribution in every layer. Each convolution layer is therefore equipped with the TCN and Bayesian inference.

The objective function of a GAN with a Wasserstein critic that replaces the discriminator part is characterized by assigning real-valued scores as shown in Equation (5), with high scores indicating the consideration of the signal as a real signal:

(5) $$\min_{G}\max_{C}\;E_{x∼p_{\mathrm{data}}}\left[C(x)\right]-E_{z∼p_{z}}\left[C(G(z))\right].$$

where

*x*$∼p_{\mathrm{data}}\left(x\right)$ is a real data sample drawn from the true data distribution;

*z*$∼p_{z}\left(z\right)$ is a latent vector from the prior;

C(·) is the critic network;

G(·) is the generator network.

The critic loss on Equation (6) is set to be minimized as its negative value is taken. Likewise, minimizing the generator loss on Equation (7) increases the critic score for the generated signals.

(6) $$L_{C}=E_{z∼p_{z}}\left[C(G(z))\right]-E_{x∼p_{\mathrm{data}}}\left[C(x)\right].$$

(7) $$L_{G}=-E_{z∼p_{z}}\left[C(G(z))\right].$$

(8) $$L_{\mathrm{rec}}={∥\hat{x}-x∥}_{2}^{2}.$$

Here,

$L_{C}$ is the critic loss;

$L_{G}$ is the generator loss;

$L_{rec}$ is the reconstruction loss.

For the critic, we use the Wasserstein objective augmented with a gradient penalty as in WGAN-GP:

(9) $$L_{C}=E_{x∼p_{\mathrm{data}}}\left[C\left(x\right)\right]-E_{z∼p_{z}}\left[C\left(G\left(z\right)\right)\right]+\lambda_{\mathrm{GP}}\;E_{\hat{x}∼p_{\hat{x}}}\left[{\left({∥\nabla_{\hat{x}}C\left(\hat{x}\right)∥}_{2}-1\right)}^{2}\right].$$

where $\hat{x}$ denotes random interpolations between real and generated samples, and $\lambda_{\mathrm{GP}}$ is the gradient-penalty weight.

This gradient penalty enforces a soft 1-Lipschitz constraint on the critic and has been shown to significantly improve training stability and sample quality compared to weight clipping in Wasserstein GANs.

The reconstruction error is considered to be one part of the losses $L_{rec}$ that contribute to the total loss of the generator. The total loss of generator is given by the sum of factored $L_{rec}$, a KL-divergence regularizer term, and the generator loss $L_{G}$ from the critic.

(10) $$L_{G,total}=\lambda_{rec}*L_{rec}+\beta_{ELBO}*L_{ELBO}+L_{G}.$$

where

$L_{G,total}$ is the total generator loss;

$\lambda_{rec}$ is the reconstruction loss weight defined based on the current and total number of epochs given by $\lambda_{\mathrm{rec}}\left(\mathrm{epoch}\right)=1+20\cdot \frac{\mathrm{epoch}}{\sqrt{\mathrm{epochs}}}$;

$\beta_{ELBO}$ is a factor that gradually increases the strength of ELBO regularization;

$L_{ELBO}$ is the ELBO loss, which is the negative of ELBO defined in Equation (4).

The encoder, generator, and critic are implemented as 1D temporal causal networks tailored to multivariate vibration signals. The deterministic encoder uses three Conv1D blocks with 64 filters and kernel size 4, with dilations 1, 2, and 4, each followed by LeakyReLU and layer normalization, and concludes with global average/max pooling and a dense layer mapping to a 64-dimensional latent vector. The conditional Bayesian generator takes this latent vector and a full wind-speed time series as inputs: the conditioning branch applies a Conv1D layer with 32 filters (kernel size 3) and global average pooling, which is concatenated with the latent vector; the main branch uses a dense layer to expand to (T×32), followed by two BayesianTCN blocks with 32 filters, kernel size 5, and dilations {1,2,4} and {4,8}, respectively, each with LeakyReLU and layer normalization, and a final Conv1D layer with kernel size 3 mapping to the original number of features with *tanh* activation.

The conditional Bayesian critic receives the input sequence concatenated with an adaptively resized wind-speed series. It begins with a BayesianTCN block with 64 filters, kernel size 4, and dilations {1,2,4,8}, followed by LeakyReLU, dropout, and layer normalization, and ends with a flatten layer and a dense output neuron. This layer-by-layer specification (filters, kernel sizes, dilations, and activations) is summarized in Table 4 and ensures that long-range dependencies and environmental conditioning are consistently modeled in both the generator and critic.

### 4.4. Adaptive Threshold

The anomaly detection framework employs a multi-component Bayesian scoring function that integrates reconstruction error, epistemic uncertainty, and critic network evaluations. The anomaly threshold in this model is computed as a weighted combination of these three components: the reconstruction error is calculated as the mean squared difference between the original sequences and their reconstructions in the validation dataset; the critic score is obtained by evaluating the realism of the generator outputs in the validation dataset using the critic network; and the epistemic uncertainty is estimated by Monte Carlo (MC) sampling of the generator to capture variability in the reconstructions [23]. Each component is then normalized and linearly combined with empirically chosen weights to produce the Bayesian combined anomaly score for a time-series sample:

(11) $$S=\alpha E_{\mathrm{norm}}+\beta U_{\mathrm{norm}}-\gamma C_{\mathrm{norm}}+\delta L_{\mathrm{norm}}-\varepsilon V_{\mathrm{norm}}.$$

where

$E_{\mathrm{norm}}$ is the normalized reconstruction error;

$U_{\mathrm{norm}}$ is the normalized epistemic uncertainty;

$C_{\mathrm{norm}}$ is the normalized critic score;

$L_{\mathrm{norm}}$ is the normalized latent-space deviation;

$V_{\mathrm{norm}}$ is the normalized variance-based penalty term;

α, β, γ, δ, and ε are weights selected via a dev-set grid search;

*S* is the combined anomaly score.

These weights are not chosen heuristically; instead, we perform a grid search on a development split of the injected-anomaly dataset and select the configuration that maximizes the F1-score under a fixed dev threshold, and then freeze the chosen weights for evaluation on the held-out test set.

The adaptive threshold is determined from the validation scores as follows:

(12) $$\tau =\mu_{\mathrm{val}}+k\cdot \sigma_{\mathrm{val}.}$$

where

$\mu_{\mathrm{val}}$ and $\sigma_{\mathrm{val}}$ are the mean and standard deviation of the combined scores computed from the validation set;

*k* is a sensitivity parameter.

A test sample is classified as anomalous if its combined score $S(x_{test})$ exceeds this threshold τ.

In practice, the validation-based threshold is defined as $\tau =\mu_{\mathrm{val}}+k\sigma_{\mathrm{val}}$, where *k* is a tunable sensitivity parameter selected on a development split rather than a fixed value. For the catenary pole case study, the weights of the combined score and the value of *k* are chosen on the dev data to maximize F1 and then frozen, and the resulting threshold is applied unchanged to the held-out test set.

The μ+kσ threshold used in this work is a simple parametric rule that assumes that the combined anomaly scores have an approximately unimodal distribution whose tails can be summarized by their mean and standard deviation. For signals or score distributions that follow mixed or strongly non-Gaussian probability laws, more robust or non-parametric thresholding strategies—such as quantile-based thresholds, mixture-model-based thresholds, or empirical false-alarm control—may be preferable. In our case study, the threshold is applied to learned anomaly scores rather than directly to raw acceleration, but we acknowledge that extending the framework to mixed-distribution settings is an important limitation and direction for future work.

## 5. Case Study

### 5.1. Dataset

Three prestressed concrete catenary poles were instrumented; the central pole M27, equipped with multiple sensors, was selected for analysis. These poles are full-scale in-service railway infrastructure elements on the Erfurt–Leipzig high-speed line and are not laboratory or scaled models. In this study, a dataset collected in 2017 was used [11]. Acceleration signals along the *x*-axis—that is, the railway direction—recorded by the sensor a12, together with the corresponding wind speed $V_{x}$ measurements from a 3D anemometer, form the dataset. Acceleration signals are used as the primary input for anomaly detection. In contrast, wind speed signals are used as a conditioning input. The dataset comprises 1606 acceleration signals, each with 114,688 timestamps. The signals are split using a 70/10/20 ratio: 1124 signals for training, 160 signals for validation, and 322 signals for testing. In this fully unsupervised approach, validation data are reserved exclusively for post-training adaptive threshold computation ($\tau =\mu_{\mathrm{val}}+k\sigma_{\mathrm{val}}$) and not used during model optimization. Synthetic anomalies are injected only into the test set for evaluation.

**Figure 5:**
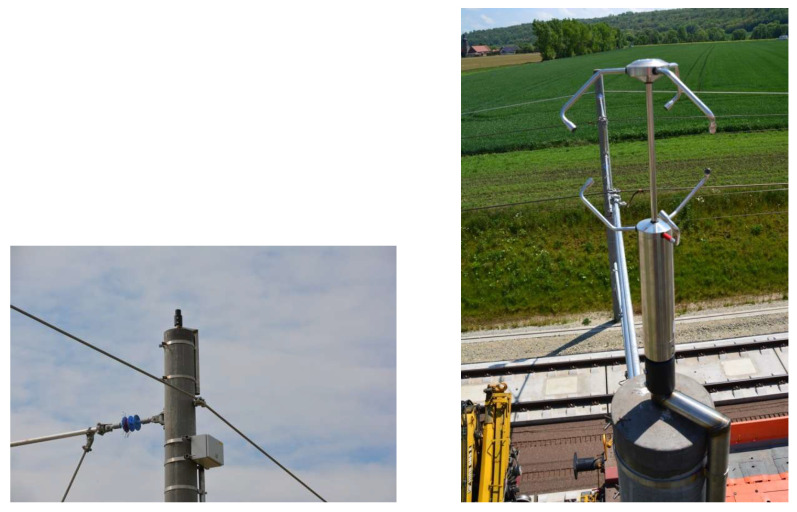
Prestressed catenary pole: (**Left**) 2D accelerometer; (**right**) 3D anemometer [25].

Figure 5 shows a prestressed catenary pole monitored with SHM sensors on the railway line between Erfurt and Leipzig, Germany [11].

Wind speed data of the same length were used as a conditioning input for both the generator and the critic. All experiments were implemented in Python 3.8.17 using TensorFlow v2.20.0, on a system with a 12th Gen Intel Core i7 processor and 32 GB RAM.

As the dataset experiences nonlinearity, KPCA served as a nonlinear dimensionality reduction tool for the accelerometer time-series dataset. This approach improved computational efficiency and model accuracy under varying operational loads.

The model was trained exclusively on healthy (normal) signals, preserving the fully unsupervised paradigm. To enable quantitative evaluation, synthetic anomalies were injected solely into the held-out test set.

The model architecture can natively incorporate the conditioning input for a desired dilation time. However, in this particular case study, it is preferred to take a reduced statistical dimension of the wind speed signal sequence expressed in 15 features in the time domain and the frequency domain. These are the mean, standard deviation, skewness, kurtosis, root mean squared, peak-to-peak, crest factor, shape factor, impulse factor, number of peaks, autocorrelation lag-1, zero-crossing, total spectral power, spectral centroid, and spectral entropy. These features effectively condense high-dimensional data into interpretable vectors, preserving patterns such as periodicity, energy distribution, and anomalies. Now each acceleration signal of n sequence has a corresponding conditional input wind signal of 15 sequences (features).

Acceleration and environmental signals were first jointly shuffled to remove ordering bias, split into train/validation/test (80%/10% of train/20%), and then standardized separately using scikit-learn’s StandardScaler (zero mean, unit variance). All sequences were downsampled to a common length of 1000 samples via mean aggregation or interpolation to match the TCN input size. Signals with insufficient variance or very low standard deviation were excluded from anomaly injection to avoid trivial or noise-dominated cases.

### 5.2. Anomaly Injection

To generate ground-truth anomalies for evaluation, we implemented a controlled injection procedure that adds subtle, structurally motivated anomalies to normal vibration signals from prestressed concrete catenary poles. The injection was applied only to the held-out test set; the training and validation sequences remained purely healthy, preserving the fully unsupervised paradigm.

First, the test signals were filtered to identify sequences with sufficient dynamic content: we computed the mean standard deviation and mean variance of each downsampled signal and retained only those above a minimum standard deviation and above the 25th variance percentile. This avoids injecting anomalies into nearly flat or noise-dominated signals. From the valid subset, a fixed fraction of signals was randomly selected for anomaly injection.

For each selected signal, we chose one or two non-overlapping time intervals subject to a minimum separation buffer and injected low-SNR anomaly patterns that reflect typical SHM mechanisms in prestressed concrete poles. The anomaly library covers slight stiffness loss or frequency softening, modeled as a subtle change in the dominant modal frequency over the interval; slight damping increase, implemented by modifying the exponential decay rate of a mode; crack breathing, represented by intermittent modulation of local stiffness via sparse on/off bursts; local prestress-loss-like ringing, where the ringing frequency changes mid-interval together with a small local step in amplitude; foundation rocking drift, modeled as a slow drift superimposed with low-frequency rocking; low-energy impacts or loosened attachments, implemented as short Gaussian-like bursts followed by weak higher-frequency ringing; weak resonance build-up, where modal amplitude gradually increases under operational excitation; and mild sensor or mounting bias, modeled as a small linear bias drift with an added high-frequency component.

Each anomaly pattern is shaped by smooth window functions and optionally mixed with band-limited noise bursts to preserve a realistic structural appearance. Patterns are scaled relative to the local signal power to achieve a target signal-to-noise ratio of 3 dB within the affected interval, ensuring that anomalies are subtle but still detectable by reconstruction and epistemic uncertainty rather than only by large amplitude spikes. Start and end indices, pattern type, and achieved SNR are recorded for each injected interval to serve as the ground truth.

**Figure 6:**
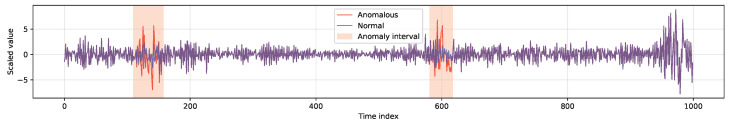
Signal with maximum SNR of 3 dB injected anomalies.

The resulting test set thus contains a mixture of purely healthy signals and signals with well-documented, low-SNR anomaly patterns that are designed to approximate early-stage damage mechanisms in prestressed concrete catenary poles Figure 6. We explicitly acknowledge that this remains a synthetic simulation; further validation on true damage events is required and is planned for future work.

Each pattern was normalized to achieve the target power, smoothed at the edges to ensure realistic transitions, and injected at non-overlapping locations within the signal. The final dataset contained normal signals and signals with damage-simulating anomalies, with the indices of the injected signals serving as the ground truth for subsequent evaluation.

### 5.3. Model Training

Following standard practice in unsupervised generative anomaly detection, the model was trained exclusively on normal data—70% of the dataset—to learn a compact representation of the normal data manifold. Leaky ReLU was used in all hidden layers across the model components to preserve gradient flow for negative activations during training. The generator’s output layer uses tanh to bound the generated signals within the same normalized range as the training data. The critic’s output layer is left without an activation function, producing an unbounded score consistent with the Wasserstein distance objective. Validation data, representing 10% of the dataset, were not used to monitor or optimize reconstruction or critic losses during training. The validation data were reserved for post-training estimation of anomaly scores, such as reconstruction- or critic-based scores, which were then used to define the data-driven anomaly detection threshold. This is typically performed by selecting a high percentage threshold, in order to preserve a tight but not overgeneralized representation of normal behavior [26,27].

The main hyperparameters used during the training and testing of the proposed model are summarized in Table 5.

As shown in Table 5, the critic network is updated twice for each generator update. This helps the critic maintain meaningful gradients, allowing the generator to learn effectively from its feedback. However, the critic should not become too strong; otherwise, the generator would receive vanishing or uninformative gradients, which would hinder its learning.

The values $\mu_{\mathrm{init}}=0.15$ and $\log\sigma_{\mathrm{init}}=1.5$ in this table specify the initialization of the BayesianTCN log-standard deviation parameters; the KL divergence is always computed against a standard normal prior N(0,1), as described in Section 4.

**Figure 7:**
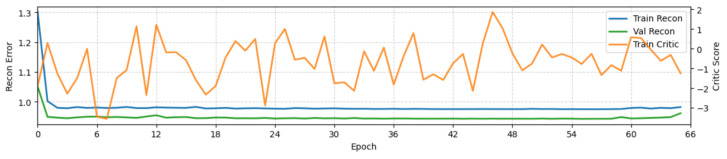
Training reconstruction error and critic score.

According to Figure 7, the training reconstruction error begins above 1.5 and decreases steadily until approximately epoch 20, after which it converges to a stable value near 1.0. This smooth decline indicates efficient initial fitting followed by stabilization as the model captures the underlying data distribution. The relatively small magnitude of the reconstruction error converging to approximately 1.0 reflects the combined effect of input data normalization and the generator’s tanh output activation, providing a normalized measure of reconstruction fidelity that establishes a stable baseline for anomaly detection. This normalization, combined with the Bayesian framework’s regularization through KL divergence, ensures that even normal signals are reconstructed with high fidelity, making deviations from this baseline more statistically meaningful for anomaly detection.

The critic score maintained a stable balance near zero throughout training, confirming the textbook Wasserstein GAN equilibrium, where neither generator nor critic dominates. After epoch 20, both metrics exhibited minimal fluctuation, indicating successful model convergence and effective manifold learning. This stability, together with the normalized error scale, provides a reliable foundation for detecting subtle damage signatures in prestressed catenary poles.

### 5.4. Latent Space Analysis

t-SNE is a nonlinear dimensionality reduction method that maps high-dimensional points into a low-dimensional space by matching probability-based similarities between pairs of points in both spaces using a Kullback–Leibler divergence objective. It preserves local neighborhoods and mitigates the crowding problem through a Gaussian kernel in the high-dimensional space and a heavy-tailed Student-t distribution in the low-dimensional space. As a result, it offers a qualitative visualization of the learned latent manifold, where compact, well-separated clusters correspond to locally similar signals and highlight how the model organizes normal and anomalous patterns [28].

**Figure 8:**
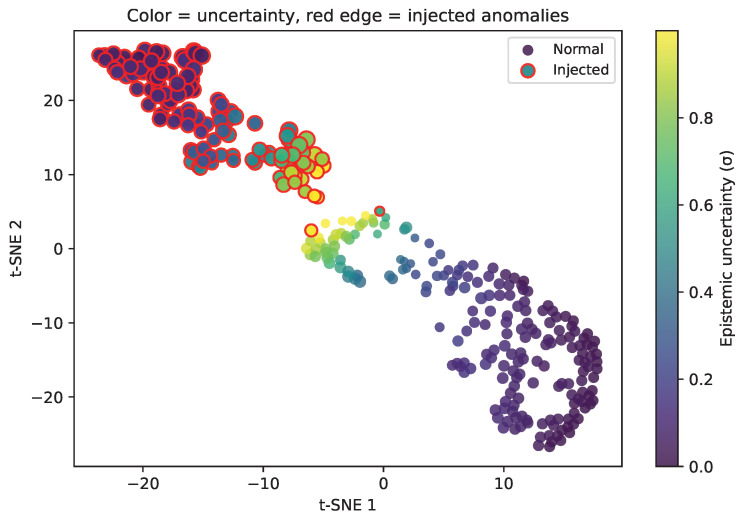
t-Distribution stochastic neighbor embedding (t-SNE).

Figure 8 shows the t-SNE visualization of the latent representations of the test set. Normal signals form a tight cluster with consistently lower epistemic uncertainty, while injected anomalies occupy a distinct region with higher epistemic uncertainty (colored by uncertainty).

Elevated uncertainty over anomalies indicates that the model has not encountered similar patterns during training, lacking confidence to classify them as normal, which is a desirable property for out-of-distribution detection. Conversely, low uncertainty over the normal cluster confirms that the encoder learns a well-defined manifold boundary, enabling reliable anomaly detection through both geometric separation and uncertainty signaling.

Beyond qualitative trends, these effects relate directly to anomaly detection performance. The reduction in posterior weight uncertainty over training is accompanied by more stable reconstruction errors and a clearer separation of the combined Bayesian score between normal and injected-anomaly signals, as reflected in the high recall, precision, F1-score, and ROC-AUC obtained on the held-out test split. The t-SNE visualization is used only as a qualitative tool to illustrate that anomalous samples tend to occupy regions of higher epistemic uncertainty in the latent space, consistent with the score distributions and the confusion matrix; quantitative conclusions are based on standard performance metrics rather than on t-SNE alone. While these trends are observed across the range of environmental conditions present in the current dataset, extending the analysis to different structures and operating regimes is an important direction for future work.

### 5.5. Validation-Data-Based Threshold and Anomaly Detection

**Figure 9:**
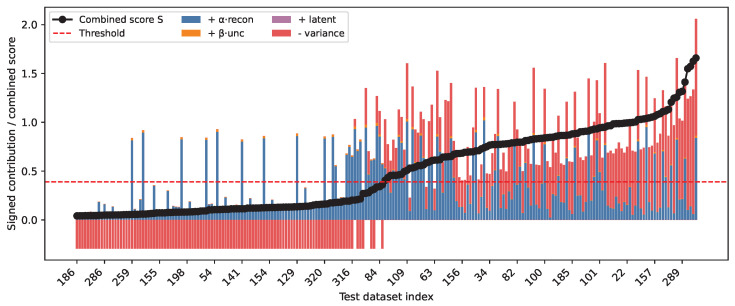
Decomposition of the combined score for the test dataset.

Figure 9 presents the decomposition of the combined anomaly score for the 322 held-out test signals (20% of the dataset), ordered by their final score values. The monotonic increase in the combined score curve and the clear separation of many samples above the threshold indicate that anomalous test signals are associated with substantially larger aggregate anomaly evidence than normal samples. The stacked contributions further show that elevated reconstruction-related and latent-space terms typically drive high-score detections, while the variance-based penalty counteracts part of this increase. Importantly, most threshold exceedances are not isolated marginal crossings but occur for samples with pronounced positive net contributions, suggesting that the final decision is supported by multiple score components rather than by small fluctuations around the threshold.

In addition to illustrating detection behavior, Figure 9 enhances interpretability by showing the component-wise contributions to the final anomaly score, which is important for trustworthy engineering AI applications [29].

**Figure 10:**
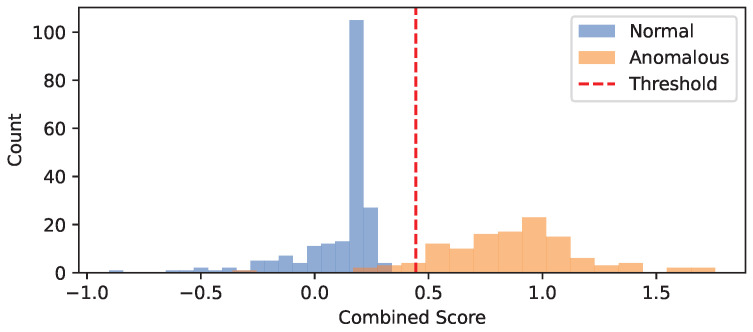
Anomaly detection with combined score distribution (log-space).

As shown in Figure 10, we first performed a grid search over 243 weight tuples on a development split of the injected-anomaly dataset and selected the best-performing combination α=0.2, β=0.2, γ=0.0, latent-space weight =0.4, and variance-penalty weight =0.4. Although Equation (10) includes a critic term $-\gamma C_{\mathrm{norm}}$, the dev-set grid search yielded γ=0.0 for the catenary pole dataset. This is physically interpretable: the injected anomalies are subtle low-SNR perturbations (SNR =3 dB) that do not deviate strongly enough from the healthy manifold to be consistently rejected by the critic, so the reconstruction error and epistemic uncertainty carry the full discriminative load. The critic term is retained in the general framework as its contribution is expected to increase for datasets with higher-SNR damage events or structurally distinct anomaly patterns.

After freezing this configuration, a validation-based threshold was computed from healthy validation scores as $\tau =\mu_{\mathrm{val}}+0.25\;\sigma_{\mathrm{val}}\approx 0.3914$. When applied unchanged to the held-out test set, this weight configuration and threshold yielded AUC=0.9848, F1=0.9587, precision =1.000, and recall =0.921, with confusion counts TP=58, FP=0, FN=5, and TN=98. In this operating regime, the model therefore achieves zero false positives on the held-out test signals, illustrating that the validation-based fusion and thresholding can be tuned to avoid false alarms while still maintaining high recall; alternative choices of *k* and weight tuples can be used if a different balance between recall and false alarms is desired in other SHM applications.

The combined score uses the five weights (α,β,γ,δ,ε) with the final values selected via the dev-set grid search. Epistemic uncertainty and reconstruction error provide the dominant contribution, while the critic term, latent-space deviation, and variance penalty refine the decision boundary. This weighting scheme yields strong separation between normal and anomalous scores and high held-out performance (ROC-AUC, F1, precision, recall), as reported above.

Of the 322 held-out test signals, 161 passed the minimum-variance eligibility filter described in Section 5.2 and were retained for quantitative evaluation; the remaining 161 signals were excluded as near-flat or noise-dominated sequences unsuitable for anomaly injection.

### 5.6. Kullback–Leibler Divergence

**Figure 11:**
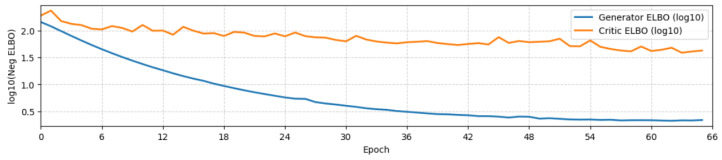
Negative ELBO minimization for generator and critic.

The training progress, visualized as the $\log_{10}$ of the negative Evidence Lower Bound (ELBO) in Figure 11, reveals distinct dynamics where the generator shows smooth and stable convergence while the critic exhibits characteristic volatility as it adapts. Crucially, because the total log-evidence is the sum of the ELBO and the Kullback–Leibler (KL) divergence, minimizing the negative ELBO, as seen in the downward trajectory of both curves, directly minimizes the KL divergence between the model’s approximation and the true data distribution. This indicates that the model is successfully closing the gap to the ground truth, resulting in more accurate generative representations.

### 5.7. Posterior Uncertainty Monte Carlo

**Figure 12:**
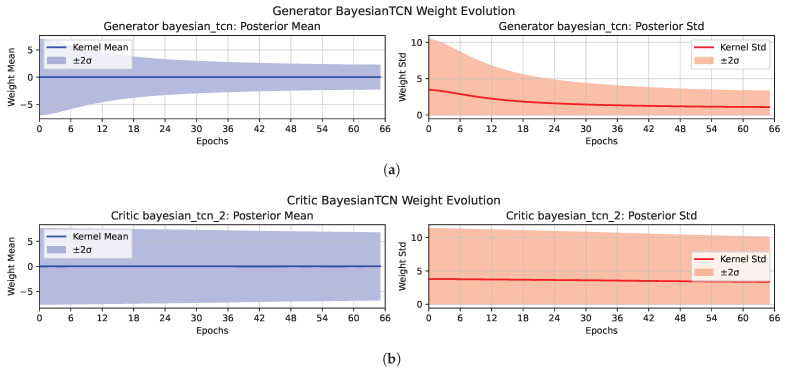
Mean and standard deviation uncertainty for generator and critic: (**a**) Generator outer layer weight evolution. (**b**) Critic outer layer weight evolution.

Figure 12 visualizes the evolution of the weight within the outer layers of the generator and critic over 100 training epochs, focusing on the posterior mean and standard deviation. In both components, the kernel mean remains exceptionally stable, centered at zero throughout the duration of training. However, the associated uncertainty regions (±2σ) exhibit a distinct contraction, particularly in the generator. Similarly, the standard deviation of the kernel for both modules shows a steady downward trajectory. The generator’s standard deviation drops from approximately 3.5 to 2.2, while the critic’s standard deviation decreases from around 4.0 to 3.0.

This simultaneous narrowing of the uncertainty bands and the reduction in standard deviation suggest a highly controlled convergence process. The stability of the mean at zero indicates that the models are not shifting their global bias but are instead concentrating their weight distributions. This statistical behavior is indicative of an effective regularization or Bayesian learning process where the model progressively prunes away stochastic noise. As training progresses, the weights transition from a broad exploratory state to a more specialized and dense configuration, effectively narrowing the search space for optimal parameters.

Ultimately, these trends signify the achievement of statistical maturity within the model. The reduction in parameter volatility across both the generator and critic points to a balanced optimization where neither component is undergoing radical, destabilizing adjustments. This tightening of the posterior distributions reflects an increase in the model’s certainty, ensuring that the final output is derived from a refined and robust set of weights.

### 5.8. Consistency of Results with Theoretical Expectations

Our Bayesian conditional GAN demonstrates strong anomaly detection performance on the dataset of prestressed concrete catenary poles. The achieved recall reflects effective learning of healthy structural dynamics from vibration data alone.

The reduction in the posterior weight uncertainty during training aligns with the theoretical predictions for Bayesian neural networks, where the epistemic uncertainty diminishes as the model observes more data, while retaining sensitivity to out-of-distribution inputs [30]. The visualization of t-SNE confirms that anomalous samples reside in regions of higher epistemic uncertainty, validating the principle that model confidence serves as a reliable indicator of input novelty in uncertainty-based deep learning frameworks [31].

The integration of Bayesian temporal causal networks within a conditional GAN architecture provides principled uncertainty quantification alongside robust temporal modeling. The conditional framework incorporates wind speed as a conditioning input, enabling the model to distinguish environmental variations from genuine structural anomalies. This uncertainty-based approach improves interpretability and reliability for safety-critical structural health monitoring applications.

## 6. Conclusions

This paper presents BcDCGAN, a Bayesian conditional deep convolutional GAN framework for unsupervised anomaly detection in the structural health monitoring of prestressed concrete catenary poles. The architecture successfully learns healthy structural dynamics from multivariate acceleration signals alone, achieving robust anomaly detection through complementary reconstruction, adversarial, and uncertainty signals.

Key contributions include variational Bayesian inference over generator and critic weights for explicit epistemic uncertainty, temporal causal networks for long-range dependency modeling, and an adaptive Bayesian scoring mechanism with data-driven thresholding. The approach demonstrates clear separation of normal and anomalous patterns in latent space, alongside appropriate uncertainty signaling, confirming effective learning of the healthy data manifold.

Experimental evaluation of real catenary pole vibration data with injected anomalies shows the potential of the methodology. High recall with interpretable uncertainty estimates supports a reliable deployment in the monitoring of critical rail infrastructure. Moreover, the component-wise anomaly scoring provides a degree of interpretability that can support practical engineering application.

Beyond the specific case study of prestressed concrete catenary poles, the proposed Bayesian conditional GAN framework can in principle be extended to more complex structural systems such as long-span bridges, building frames, and multi-support viaducts. However, scalability to such systems introduces additional challenges, including richer modal interactions, more heterogeneous operating conditions, and the need for more diverse and representative healthy training data. The computational cost of Bayesian inference also increases with network size and the number of sensor channels. These factors may affect model performance and reliability if not properly addressed.

Future work will therefore focus on multi-sensor fusion, transfer learning across structures, and model simplification strategies to maintain tractable uncertainty quantification while scaling to larger, more complex civil infrastructure.

Despite the promising results, the proposed framework has several limitations: First, the evaluation relies on synthetically injected anomalies rather than confirmed field damage cases, which may not fully capture the variability and complexity of real structural degradation. Second, the final anomaly decision depends on score-component weights and a validation-based threshold, both of which may require recalibration under different datasets, structures, or environmental conditions. Third, the current study is based on one catenary pole monitoring scenario, and broader validation across multiple assets and operating regimes is still needed. Finally, although the Bayesian formulation provides uncertainty estimates, the overall model remains computationally more demanding than simpler unsupervised baselines.

Future work will extend validation to natural damage scenarios and investigate multi-sensor fusion across catenary systems. Real-time implementation and transfer learning to other civil infrastructure will further enhance the framework’s practical impact for structural health monitoring.

## Figures and Tables

**Table 1:** Baseline anomaly detection performance on the catenary pole test set with injected anomalies. All thresholds use a μ+kσ rule estimated from healthy validation data and applied unchanged to the held-out test signals.

Method	TP	FP	FN	TN	Precision	Recall	F1	ROC-AUC
RMS + μ+kσ (statistical)	32	31	88	171	0.51	0.27	0.35	0.70
KPCA reconstruction error	21	20	99	182	0.51	0.18	0.26	0.70
CNN/TCN autoencoder	19	12	101	190	0.61	0.16	0.25	0.70

**Table 2:** GAN-based anomaly detection approaches.

Method	How It Works	Strength	Limitation
TAnoGAN	GAN with LSTM, uses reconstruction errors	Models temporal trends	Tuning sensitive
DCGAN + Bi-LSTM	DCGAN and Bi-LSTM for spatial–temporal data	Accurate for sequences	Computationally heavy
BiGAN	Joint encoder, generator, discriminator training	Precise reconstruction	Overfitting risk

**Table 3:** Evaluation metrics.

Strategy/Metric	Description
Reconstruction Error	MSE or similar between input and reconstruction
Discriminator Score	Confidence near 0.5 indicates uncertainty
Combined Scoring	Fusion of residual and discriminative signals
Thresholding Approaches	Fixed, percentile, or μ+kσ
Recall	TP/(TP + FN) on injected anomalies
Precision, F1 and F2	Trade-off between missed faults and false alarms

**Table 4:** Layer-by-layer components of the BcDCGAN architecture.

(A) Deterministic Encoder					
Layer/Block	Role	Filters	Kernel	Dilation	Activation/Norm
Input	Acceleration signal	–	–	–	–
Conv1D-1	TCN Block (det.)	64	4	1	LeakyReLU + LayerNorm
Conv1D-2	TCN Block (det.)	64	4	2	LeakyReLU + LayerNorm
Conv1D-3	TCN Block (det.)	64	4	4	LeakyReLU + LayerNorm
GlobalPool	Avg + Max Pooling	–	–	–	–
Dense	Latent projection	–	–	–	Linear
**(B) Conditional Bayesian Generator**					
**Layer/Block**	**Role**	**Filters**	**Kernel**	**Dilation**	**Activation/Norm**
Input	Latent *z* + wind cond.	–	–	–	–
Cond. Branch	Conv1D (wind features)	32	3	1	GlobalAvgPool
Dense	Expand latent to T×32	–	–	–	Linear
B-TCN Block 1	Bayesian TCN Block	32	5	{1,2,4}	LeakyReLU + LayerNorm
B-TCN Block 2	Bayesian TCN Block	32	5	{4,8}	LeakyReLU + LayerNorm
Conv1D (out)	Output projection	1	3	1	tanh
**(C) Conditional Bayesian Critic**					
**Layer/Block**	**Role**	**Filters**	**Kernel**	**Dilation**	**Activation/Norm**
Input	Signal + wind (concat.)	–	–	–	–
B-TCN Block	Bayesian TCN Block	64	4	{1,2,4,8}	LeakyReLU + Dropout + LayerNorm
Flatten	Flatten	–	–	–	–
Dense (out)	WGAN-GP score	1	–	–	Linear (none)

**Table 5:** Key hyperparameters used for training the Bayesian conditional DCGAN.

Category	Parameter	Description
Training setup	E=66	Total number of training epochs.
	$n_{C}=2$	Number of critic updates per generator/ encoder update.
Optimizers	Adam (G, E), $\eta =5\times 10^{-3},\;\beta_{1}=0.9$	Optimizer and hyperparameters for generator and encoder.
	Adam (C), $\eta =1\times 10^{-3},\;\beta_{1}=0.5$	Optimizer and hyperparameters for critic.
Posterior init.	$\mu_{\mathrm{init}}=0.15$	Initial mean of the variational posterior q(θ) for Bayesian TCN weights; KL divergence is computed against the fixed prior p(θ)=N(0,1).
	$\log\sigma_{\mathrm{init}}=1.5$	Initial log-std of the variational posterior q(θ) for Bayesian TCN weights.
ELBO regularization	$\beta_{\mathrm{ELBO}}=0.9$	Target weight on the ELBO-based regularization term.
Uncertainty	$N_{\mathrm{MC}}=80$	Number of Monte Carlo forward passes per input to estimate epistemic uncertainty.
Anomaly scoring	(α,β,γ,δ,ε)	Weights for (reconstruction, uncertainty, critic, latent, variance) in the combined log-space anomaly score, selected via dev-set grid search.
Thresholding	$\tau =\mu_{\mathrm{val}}+k\sigma_{\mathrm{val}},\;k=0.25$	Adaptive validation-based anomaly threshold.

## Data Availability

The data presented in this study are available within the article.
